# Supplementary material for: Characteristics, experiences and actions taken by women to address delayed conception: A mixed-methods cross-sectional study protocol
Source: PLoS One. 2022 Mar 11;17(3):e0264777. doi: 10.1371/journal.pone.0264777 (PMC8916660; doi:10.1371/journal.pone.0264777)
Supplement: S3 File — (PDF) [file pone.0264777.s003.pdf]

PARTICIPANT INFORMATION SHEET AND INFORMED CONSENT FORM

To be administered to potentially eligible women enrolled in WINGS who have completed 18 months and are not pregnant

Baseline characteristics, experiences and actions taken by women to address failure to conceive, among women who exit WINGS without getting pregnant

INVESTIGATORS

Centre for Health Research and Development, Society for Applied Studies (CHRD-SAS)

Sarmila Mazumder, Neeta Dhabhai, Sunita Taneja, Ranadip Chowdhury and Nita Bhandari

Summary

Greetings! My name is ________________________. I am from the Centre for Health Research and Development, Society for Applied Studies. We are contacting you as you had agreed to be contacted for the new study. We are requesting you to participate in a research study which is being conducted by us and is supported by World Health Organization (WHO). The purpose of this document is to help you decide your participation in the study and give your consent for participation if you agree to do so.

Please read this document carefully or we can read it to you. This Information Sheet contains information about the study on infertility. We are asking you to read (or have read to you) this consent form. This is to make sure that you are informed about being in this study. You will be asked to sign this consent form. We will give you a copy of this form or just a sheet with contact information on it . This consent form might contain some words that are unfamiliar to you. Please ask us to explain anything you may not understand.

If you feel, you may keep an unsigned copy of this document so that you can think and discuss with your family about participating in the study. You should not agree to participation till all your questions are answered by us.

If you decide to take part in the study, you will be given a copy of the signed document.

Purpose of the study

In India around 4 to 17% of couples are estimated to have difficulty in conceiving. There are many reasons why couples may struggle to conceive. In some cases, conceiving is just a matter of time, and in due course, couples conceive. In other cases, there might be a medical reason for delay in conceiving. These reasons could be related to the woman, her body parts that are involved in bearing a child, for example, producing eggs, or the state of her womb, or her hormones. However the reasons can also be related to the man and his body mechanisms that are needed to produce sperms to form a baby. In both men and women infections and certain medications can make conceiving difficult.

Difficulty to conceive and bear a child, has significant social and public health significance. The ability to bear a biological child is deemed to indicate a woman's health, and consequently, her position in society. Difficulties in conceiving is an important source of emotional and psychological distress among affected couples, who are often stigmatised. Research focussing on infertility has primarily shown the importance of STIs and associated pelvic inflammation on female reproductive organs.

At the same time, understanding how women who experience difficulties in conceiving cope with their circumstances is an essential first step towards providing them with necessary supportive interventions.

You have been a part of the WINGS and completed 18 months without becoming pregnant. This is a new study and your participation is entirely voluntary and not obligatory. In this study we would like to find out how women who are waiting to get pregnant experience the period of waiting, how they cope with the waiting, and what kind of actions they and their husbands take to increase their chances of getting pregnant. The results of this study will be used to plan health interventions to assist women who may be experiencing difficulties in conceiving a child and to determine how best to support them in India.

Procedures

We are approaching you to participate in this study. Your participation is entirely voluntary and not obligatory. If you do not agree to participate in this study, there will be no implications or consequences to you or your family, whatsoever. It is completely up to your decision to participate or not participate.

If you agree to be in this research and if you have been waiting to conceive for 18 months, we will ask you some questions on age, education, occupation, income of the family, religion, family type, family members, source of drinking water, place of defecation, housing structure, religious and cultural beliefs around infertility, overall health and reproductive health related issues, your experience with getting treatment for certain symptoms, sexual history, about your plans to get a child and previous pregnancy. We will also find out from you if you have other symptoms related to sexual infections. Any information you give will be confidential. You have the right to refuse to answer any questions etc. Some of the women will be included in a follow up group discussion to discuss this issue with other women in a similar situation. If you are selected for the group discussion, we will invite you to participate. This will take place at a later point. You don't have to participate in the group discussion. You may choose to only participate in answering our questions without going on to participate in the group discussion with other women.

Your part in this research will take between one and a half to two hours to respond to our questions. If you are selected and would be interested to participate in the group discussion, you would need an additional hour for the discussion. We expect 1530 women to take part in the survey, and 50 women to participate in the group discussions.

The group discussions will be held in your preferred language and will be audio recorded to allow the researchers to listen to the conversations later. We will not record your name on any of the questionnaire or the audio recorded discussions. These data will only be labeled with your anonymous study number.

Benefits

There is no direct benefit to you from participating in this study. However, the whole community of women who are experiencing delays in conceiving may benefit if interventions and support are provided to such women as a result of this study. We will also facilitate your referral to infertility clinic in Safdarjung Hospital, if you are willing to visit.

Risk and Discomforts

There is a chance that some of the questions asked may make you feel uncomfortable psychologically. The time taken to collect information may be inconvenient for you. There is some limited social risk of being identified by others when you participate in the study and focus group discussions, but we will make sure that your participation and that of the group is anonymous and done in private. We will not record your name in any study documents and audio records. All documents will be kept in locked files. Results of the study will be released appropriately and after consultation with health officials and community leaders. At any time, you may refuse to answer any questions. You may also withdraw from the study at any time.

If you decide not to be in the research

You are free to decide if you want to be in this research. Your decision will not affect the health care you would normally receive.

Confidentiality

The information collected from you will be kept confidential. You will not be identified by your name but only by a study number. All information collected will be stored in a locked area with access only to the study team. All study documents will be stored for a period of 5 years. The knowledge which we get from doing this research study will be shared through reports given to the ethics committee, government agencies and publications but none will have your name.

Potential secondary use of your data in future

We may need to use the data you give us to address a different research question in the future. We do not know precisely what future research questions would be. In the event that we conduct further analysis of the information you give us, we will protect information about you and your taking part in this research to the best of our ability.

Voluntary participation and withdrawal

Taking part in this study is voluntary. You may decide not to participate or you may choose to leave the study anytime. Your decision will not affect access to any services that you are currently receiving from any providers.

Cost and payment for participation

You will neither get any monetary benefit nor will you have to bear any cost for participating in this study. However you will receive some small utility items in appreciation for your time.

Who to Contact

If you have any questions related to the study, you can ask them now. If you want, you can speak to the following persons:

Sarmila Mazumder: 9811681530, Neeta Dhabhai: 9811268495, Sunita Taneja: 9811206456, Ranadip Chowdhury: 9836685913

Your rights as a participant

This research has been reviewed and approved by the Ethical Review Committee. This is a committee that reviews research studies in order to help protect the participant.

If you have any questions related to the conduct of the study or have any questions related to your rights as a participant, you can contact the Ethics Review Committee. The contact number is 7838350052.

Baseline characteristics, experiences and actions taken by women to address failure to conceive, among women who exit WINGS without getting pregnant

INFORMED CONSENT FORM FOR ENROLMENT INTO THE STUDY

Name: ___________________________________

Women ID: _______________________________

	**Write Yes or No**
(i) I confirm that I have read the information provided or have had it read. I had the opportunity to ask questions and any questions that I have asked have been answered to my satisfaction and I consent voluntarily to participate in this research.	________
(ii) I confirm that I have understood the procedures of the study and what is expected of me	________
(iii) I understand that my participation in the study is voluntary and that I am free to withdraw at any time, without giving any reason, without my access to routine facilities from my preferred service providers or rights being affected.	________
(iv) I understand that the Ethics Committees and study team will not need my permission to look at my records collected during the study, even if I withdraw myself from the study. I agree to this access. However, I understand that my identity will not be revealed in any report or publication	________
(v) I agree to the future use of any data collected during this study, provided that the data are used only for scientific purpose(s)	________
(vi) I agree for the interview	________
(vii) I agree to participate in the group discussion with audio recording	________
(vii) I agree to participate in the group discussion without audio recording	________

Name of Participant__________________

Signature of Participant ____________________

Date ____________________________

DD/MMM/YYYY

Statement by the witness (If participant not-literate)

I have witnessed the accurate reading of the information sheet to the potential participant, and she has had the opportunity to ask questions and her questions have been answered to her satisfaction. I confirm that she has given consent freely and that the woman is willing to participate in the research study.

Name of the participant__________________________ **Thumb impression of participant**

Name of witness______________________

Signature of witness ______________________

Date _________________________

DD/MMM/YYYY

Statement by the person administering consent

I confirm that the woman has read/I have read out the information sheet to the woman. I confirm that she has understood the procedures explained, she was given an opportunity to ask questions about the research study, and all the questions asked by her have been answered correctly. She has not been coerced and has given consent voluntarily.
A signed copy of this consent form along with the information sheet is being provided to the participant.

Name of person administering the consent_________________________

Signature of the person administering the consent ___________________

Date _________________________

DD/MMM/YYYY
