# Supplementary material for: Characteristics, experiences and actions taken by women to address delayed conception: A mixed-methods cross-sectional study protocol
Source: PLoS One. 2022 Mar 11;17(3):e0264777. doi: 10.1371/journal.pone.0264777 (PMC8916660; doi:10.1371/journal.pone.0264777)
Supplement: S1 Questionnaire — (PDF) [file pone.0264777.s004.pdf]

अध्ययन के दौरान गर्भवती न होने के कारण विंगस से बाहर हुई महिलाओं की आधारभूत विशेषताएं, गर्भधारण में सफल न होने के संबंध मे महिलाओं द्वारा किये गये अनुभव और उपाय।

Infertility Study ID: ☐☐☐☐☐☐☐☐☐☐

MODULE 1: GENERAL HISTORY AND FERTILITY INTENTIONS

मॉडयूल 1: सामान्य इतिहास और प्रजनन का इरादा

Administer this questionnaire to women who did not conceive in the 18 months of follow up in WINGS

इस प्रश्नावली को उन महिलाओं के लिए भरा जाएगा जो पिछले 18 महिनों में विंगस में फॅलो–अप करते समय गर्भवती नही हुई

[Code: 1=Yes, 2=No, 8=Does not know, 9=Not Applicable]
[कोडः 1=हॉं, 2=नहीं, 8=पता नहीं, 9=मान्य नहीं]

General History and Socio-demographic Data. [The questions on sociodemographic status will not be repeated if the same woman is enrolled in multiple studies]
सामान्य इतिहास और सामाजिक–जनसंख्या के आंकड़े (सामाजिक–जनसंख्या स्थिति संबंधित परेशानी दोहराए नही जाएंगी अगर यही औरत/महीला कोई और परियोजना में भाग लिया हो)

1.	WINGS ID<br>विंगस आई.डी.	1.	☐☐☐☐☐☐
2.	Respondent’s study ID number (eg INFERT0001 TO INFERT1530)<br>उतरदेने वाले का स्टडी आई.डी. नम्बर (eg INFERT0001 TO INFERT1530)	2.	☐☐☐☐☐☐☐☐☐☐
3.	Questionnaire administered by प्रश्नावली किसके द्वारा भरा गया	3.	____________________ ☐☐
4.	Consent obtained? सहमति प्राप्ती हुई ?	4.	☐
5.	Date तारीख	5.	☐☐☐☐☐☐☐☐
**Partnership and children circumstances**			
6.	What is your age?<br>आपकी उम्र क्या हैं ?	6.	☐☐
7.	What is your marital status (11= Married, 12=single, 13= Divorced, 14= Widowed)	7.	☐☐

अध्ययन के दौरान गर्भवती न होने के कारण विंगस से बाहर हुई महिलाओं की आधारभूत विशेषताएं, गर्भधारण में सफल न होने के संबंध मे महिलाओं द्वारा किये गये अनुभव और उपाय।

Infertility Study ID: ☐☐☐☐☐☐☐☐☐☐

	आपकी वर्तमान विवाहिक स्थिति क्या हैं (11= शादीशुदा, 12= अविवाहित, 13= तलाकशुदा, 14=विधवा)		
8.	Have you ever had a child?<br>क्या आपने कभी किसी बच्चे को जन्म दिया हैं ?	8.	☐
9.	If YES, what was your age at the birth of first child?<br>अगर हाँ तो जब आपने आपके पहले बच्चे को जन्म दिया था तब कितने साल की थी ?	9.	☐☐
10.	Date when you last delivered?<br>पिछ्ले प्रसव की तारीख क्या थी ?	10.	☐☐☐☐☐☐☐☐
11.	How many children do you intend to have in life?<br>आप कितने बच्चे चाहते है ?	11.	☐☐
12.	Do you have any adopted, fostered or step children?<br>क्या आपके कोई गोद लिया हुआ या सौतेले बच्चे है ?	12.	☐
13.	What is your husband's age?<br>आपके पति कितने साल के हैं ?	13.	☐☐
14.	How many years have you been married with your husband?<br>कितने सालों से आप और आपके पति शादीशुदा है ?	14.	☐☐
15.	Has your husband ever fathered any children?<br>क्या आपके पति कोई संतान के पिता बने है ?	15.	☐
**Wanting pregnancy/ Fertility intentions**			
16.	Had you been trying to get pregnant prior to joining WINGS? (YES/NO)<br>जिस परियोजना मे आप 1½ साल से शामिल थी उस परियोजना में नाम लिखाने से पहले क्या आप गर्भवती होने की कोशिश कर रही थी ?	16.	☐

अध्ययन के दौरान गर्भवती न होने के कारण विंगस से बाहर हुई महिलाओं की आधारभूत विशेषताएं, गर्भधारण में सफल न होने के संबंध मे महिलाओं द्वारा किये गये अनुभव और उपाय।

Infertility Study ID: ☐☐☐☐☐☐☐☐☐☐

17.	In total, how long have you been trying to conceive (in Years)<br>कुल मिलाकर कितने समय से आप गर्भवती होने की कोशिश कर रही है (सालों में बताएं)	17.	☐☐
18.	When you joined WINGS did you really want to become pregnant?<br>जब आप उस परियोजना मे शामिल हुए थे, क्या तब आप सच में गर्भवती होना चाहती थी ?	18.	☐
19.	Have you been trying to get pregnant all the time you have been in the study?<br>उस परियोजना मे शामिल होने के दौरान क्या आप लगातार गर्भवती होने की कोशिश कर रही थी ?	19.	☐
20.	How frequently have you been having unprotected vaginal intercourse?<br>कितनी बार आपने पति से संबंध बनाया बिना कोई प्रोटेक्शन/सेफटी लिए ?	20.	
20.1	Once every month महीने में एक बार	20.1	☐
20.2	Twice a month महीने में दो बार	20.2	☐
20.3	Once a week हफतें में एक बार	20.3	☐
20.4	Twice a week हफतें में दो बार	20.4	☐
20.5	More than twice a week हफतें में दो बार से ज्यादा	20.5	☐
21.	If No, for how many months were you not trying to become pregnant? (in Months)<br>अगर नहीं, उस परियोजना मे शामिल होने के दौरान कितने समय तक आपने गर्भवती होने की कोशिश नही की ? (महीनें में बताएं)	21.	☐☐
22.	Do you feel that you were taking too long to become pregnant?	22.	☐

अध्ययन के दौरान गर्भवती न होने के कारण विंगस से बाहर हुई महिलाओं की आधारभूत विशेषताएं, गर्भधारण में सफल न होने के संबंध मे महिलाओं द्वारा किये गये अनुभव और उपाय।

Infertility Study ID: ☐☐☐☐☐☐☐☐☐☐

	क्या आपको लगता है कि आप बहुत लम्बा समय ले रही थी गर्भवती होने के लिए ?		
23.	Has your husband told you that you have taken too long to become pregnant?<br>क्या आपके पति ने आपको कहा हैं कि आप बहुत समय ले चुके हैं गर्भवती होने के लिए ?	23.	☐
24.	Have you felt isolated, discriminated or stigmatized because you were taking too long to become pregnant?<br>क्या आपने कभी महसूस किया हैं कि आपको अलग किया जा रहा हैं समाज से या आपके साथ भेदभाव हो रहा है या आप लांछित हो रही हैं क्योंकि आप गर्भवती होने में ज्यादा समय ले रही हैं ?	24.	☐
25.	Have you experienced any of the following from your husband because you were taking too long to become pregnant?<br>क्या इस कारण आपने कभी आपके पति से कुछ ऐसे बर्ताव का अनुभव किया हैंः	25.	
25.1	Physical abuse शारिरिक अत्याचार	25.1	☐
25.2	Emotional abuse मानसिक अत्याचार	25.2	☐
25.3	Verbal abuse गाली–गलौच	25.3	☐
25.4	Denial of financial support आर्थिक सहायता से वंचित	25.4	☐
25.5	Divorce तलाक	25.5	☐
25.6	Abandonment परित्याग	25.6	☐
25.7	Fathering a child with another woman अन्य महिला के साथ बच्चे को जन्म दिया	25.7	☐

अध्ययन के दौरान गर्भवती न होने के कारण विंगस से बाहर हुई महिलाओं की आधारभूत विशेषताएं, गर्भधारण में सफल न होने के संबंध मे महिलाओं द्वारा किये गये अनुभव और उपाय।

Infertility Study ID: ☐☐☐☐☐☐☐☐☐☐

26.	Have you experienced any of the following from anyone else in your family, because you were taking too long to become pregnant?<br>क्या आपने कभी आपके परिवार के किसी और सदस्य से इस लिस्ट से कुछ भी अनुभव किया हैं क्योंकि आप बहुत लम्बा समय ले रही थी गर्भवती होने के लिए ?	26.	
26.1	Physical abuse शारिरिक अत्याचार	26.1	☐
26.2	Emotional abuse मानसिक अत्याचार	26.2	☐
26.3	Verbal abuse गाली–गलौच	26.3	☐
26.4	Denial of financial support आर्थिक सहायता से वंचित	26.4	☐
26.5	Divorce तलाक	26.5	☐
26.6	Abandonment परित्याग	26.6	☐
26.7	Fathering a child with another woman अन्य महिला के साथ बच्चे को जन्म दिया	26.7	☐
27.	If yes, from whom<br>अगर हाँ है तो कैसे आपको यह अनुभव हुआ	27.	
27.1	Mother-in-law सास	27.1	☐
27.2	Sister-in-law ननद	27.2	☐
27.3	Father-in-law ससुर	27.3	☐
27.4	Brother-in-law देवर	27.4	☐
27.5	Others अन्य कोई नहीं	27.5	☐

अध्ययन के दौरान गर्भवती न होने के कारण विंगस से बाहर हुई महिलाओं की आधारभूत विशेषताएं, गर्भधारण में सफल न होने के संबंध मे महिलाओं द्वारा किये गये अनुभव और उपाय।

Infertility Study ID: ☐☐☐☐☐☐☐☐☐☐

Form C: Module 2

Administer this questionnaire to women who did not conceive in the 18 months of follow up in WINGS

मॉडयूल 2

इस प्रश्नावली को उन महिलाओं के लिए भरा जाएगा जो पिछले 18 महिनों में विंगस में फॅलो–अप करते समय गर्भवती नही हुई

[Code: 1=Yes, 2=No, 8=Does not know, 9=Not Applicable]
[कोडः 1=हॉं, 2=नहीं, 8=पता नहीं, 9=मान्य नहीं**]**

1.	WINGS ID<br>विंगस आई.डी.	1.	☐☐☐☐☐☐
2.	Respondent's study ID number (eg INFERT0001 TO INFERT1530)<br>उतरदेने वाले का स्टडी आई.डी. नम्बर (eg INFERT0001 TO INFERT1530)	2.	☐☐☐☐☐☐☐☐☐☐
3.	Questionnaire administered by प्रश्नावली किसके द्वारा भरा गया	3.	____________________ ☐☐
4.	Consent obtained? सहमति प्राप्ती हुई ?	4.	☐
5.	Date तारीख	5.	☐☐☐☐☐☐☐☐
**MODULE 2: QUALITY OF LIFE** (FERTQoL) जीवन की गुनवक्ता (जीवन की प्रजननक्षमता की गुनवक्ता संबंधित परेशानियॉं)			
6.	How would you rate your health?<br>आप अपने स्वास्थ्य को किस तरह मूलायांकन करेंगें (आप अपने स्वास्थ्य के बारे में क्या सोचते हैं) ?<br>(3= Very poor; 4= poor; 5= Neither good nor bad; 6= Good; 7= Very good)<br>(3=बहुत खराब, 4=खराब, 5=ना अच्छा ना खराब, 6=अच्छा, 7=बहुत अच्छा)	6.	☐

अध्ययन के दौरान गर्भवती न होने के कारण विंगस से बाहर हुई महिलाओं की आधारभूत विशेषताएं, गर्भधारण में सफल न होने के संबंध मे महिलाओं द्वारा किये गये अनुभव और उपाय।

Infertility Study ID: ☐☐☐☐☐☐☐☐☐☐

7.	Are you satisfied with your quality of life?<br>(3= Very dissatisfied; 4= Dissatisfied; 5= Neither satisfied nor dissatisfied; 6= Satisfied; 7= Very satisfied)<br>आपके जीवन में शारीरिक खुशहाली, मानसिक खुशहाली और सामाजिक खुशहाली से क्या आप संतुष्ट हैं?<br>(3=बहुत असंतुष्ट, 4=असंतुष्ट, 5=ना संतुष्ट ना असंतुष्ट, 6=संतुष्ट, 7=बहुत संतुष्ट)	7.	☐
8.	Do you think you cannot move ahead with other life goals and plans because of fertility problems?<br>(3= Completely, 4= A great deal; 5= Moderately; 6= Not much; 7= Not at all)<br>क्या आपको लगता हैं कि जो बच्चा होने मे समय लग रहा है उसके कारण जीवन में जो बाकी लक्ष्य है, और उसके लिए जो काम आपको करना हैं वह रूक सा गया हैं?<br>(3=पूरी तरह, 4=काफी हद तक, 5=ठीक-ठीक, 6=ज्यादा नहीं, 7=बिल्कुल नहीं)	8.	☐
9.	Do you feel drained or worn out because of fertility problems?<br>(3= Completely, 4= A great deal; 5= Moderately; 6= Not much; 7= Not at all)<br>क्या आप अपनी इस (प्रजननक्षमता की) समस्या के कारण अपने आपको थका हुआ या टूटा हुआ महसूस करते हैं?<br>(3=पूरी तरह, 4=काफी हद तक, 5=ठीक-ठीक, 6=ज्यादा नहीं, 7=बिल्कुल नहीं)	9.	☐
10.	Do you feel able to cope with your fertility problems?<br>(3= Completely, 4= A great deal; 5= Moderately; 6= Not much; 7= Not at all)	10.	☐

अध्ययन के दौरान गर्भवती न होने के कारण विंगस से बाहर हुई महिलाओं की आधारभूत विशेषताएं, गर्भधारण में सफल न होने के संबंध मे महिलाओं द्वारा किये गये अनुभव और उपाय।

Infertility Study ID: ☐☐☐☐☐☐☐☐☐☐

	क्या आपको लगता हैं कि आप अपनी इस (प्रजननक्षमता की) समस्या का सामना कर पाते हैं ?<br>(3=पूरी तरह, 4=काफी हद तक, 5=ठीक–ठीक, 6=ज्यादा नहीं, 7=बिल्कुल नहीं)		
11.	Are you satisfied with the support you receive from friends with regard to your fertility problems?<br>(3= Very dissatisfied; 4= Dissatisfied; 5= Neither satisfied nor dissatisfied; 6= Satisfied; 7= Very satisfied)<br>इस (प्रजननक्षमता की) समस्या के बारे में जो सहयोग मिलता है दोस्तों से, क्या आप उससे संतुष्ट हैं ?<br>(3=बहुत असंतुष्ट, 4=असंतुष्ट, 5=ना संतुष्ट ना असंतुष्ट, 6=संतुष्ट, 7=बहुत संतुष्ट)	11.	☐
12.	Are you satisfied with your sexual relationship even though you have fertility problems?<br>(3= Very dissatisfied; 4= Dissatisfied; 5= Neither satisfied nor dissatisfied; 6= Satisfied; 7= Very satisfied)<br>इस (प्रजननक्षमता की) समस्या के बावजूद अपनी यौन संबंधों (पति से शारिरीक संबंध) से आप संतुष्ट हैं ?<br>(3=बहुत असंतुष्ट, 4=असंतुष्ट, 5=ना संतुष्ट ना असंतुष्ट, 6=संतुष्ट, 7=बहुत संतुष्ट)	12.	☐
13.	Do your fertility problems cause feelings of jealousy and resentment?<br>(3= Always; 4= Very often; 5= Quite often; 6= Seldom; 7= Never)<br>क्या अपनी इस (प्रजननक्षमता की) समस्या आपको ईशा/जलन या नाराज़गी/गुस्सा महसूस कराती हैं ?	13.	☐

अध्ययन के दौरान गर्भवती न होने के कारण विंगस से बाहर हुई महिलाओं की आधारभूत विशेषताएं, गर्भधारण में सफल न होने के संबंध मे महिलाओं द्वारा किये गये अनुभव और उपाय।

Infertility Study ID: □□□□□□□□□□

	(3=हमेशा, 4=बहुत बार, 5=अक्सर, 6=कभी–कभी, 7=कभी नहीं)		
14.	Do you experience grief and/or feelings of loss about not being able to have a child (or more children)?<br>(3= Always; 4= Very often; 5= Quite often; 6= Seldom; 7= Never)<br>क्या आप बच्चा या और अधिक बच्चे ना कर पाने की स्थिति के बारे में दुख तथा अपने जीवन में किसी कमी का अनुभव करती हैं?<br>(3=हमेशा, 4=बहुत बार, 5=अक्सर, 6=कभी–कभी, 7=कभी नहीं)	14.	□
15.	Do you fluctuate between hope and despair because of fertility problems?<br>(3= Always; 4= Very often; 5= Quite often; 6= Seldom; 7= Never)<br>इस (प्रजननक्षमता की) समस्या होने से क्या आपकी भावनाओं, आशा और निराशा के बीच उतार–चढ़ाव करती हैं?<br>(3=हमेशा, 4=बहुत बार, 5=अक्सर, 6=कभी–कभी, 7=कभी नहीं)	15.	□
16.	Are you socially isolated because of fertility problems?<br>(3= Always; 4= Very often; 5= Quite often; 6= Seldom; 7= Never)<br>क्या आप इस (प्रजननक्षमता की) समस्या के कारण सामाजिक रूप से अलग–थलग हैं?<br>(3=हमेशा, 4=बहुत बार, 5=अक्सर, 6=कभी–कभी, 7=कभी नहीं)	16.	□
17.	Are you and your husband affectionate with each other even though you have fertility problems?<br>(3= Always; 4= Very often; 5= Quite often; 6= Seldom; 7= Never)	17.	□

अध्ययन के दौरान गर्भवती न होने के कारण विंगस से बाहर हुई महिलाओं की आधारभूत विशेषताएं, गर्भधारण में सफल न होने के संबंध मे महिलाओं द्वारा किये गये अनुभव और उपाय।

Infertility Study ID: ☐☐☐☐☐☐☐☐☐☐

	इस समस्या के बावजूद क्या आप और आपके पति एक दूसरे से स्नेह/प्यार करते है? (3=हमेशा, 4=बहुत बार, 5=अक्सर, 6=कभी–कभी, 7=कभी नहीं)		
18.	Do your fertility problems interfere with your day-to-day work or obligations? (3= Always; 4= Very often; 5= Quite often; 6= Seldom; 7= Never) क्या आपकी इस (प्रजननक्षमता की) समस्या संबंधी समस्यॉं आपके रोजमर्रा के कार्यो या जिम्मेदारियों पर असर डालती है? (3=हमेशा, 4=बहुत बार, 5=अक्सर, 6=कभी–कभी, 7=कभी नहीं)	18.	☐
19.	Do you feel uncomfortable attending social situations like holidays and celebrations because of your fertility problems? (3= Always; 4= Very often; 5= Quite often; 6= Seldom; 7= Never) इस (प्रजननक्षमता की) समस्या की वज़ह से क्या आप छूट्टियॉं या उत्सव जैसे सामाजिक परिस्थितियों में भाग लेने में असुविधा महसूस करती है? (3=हमेशा, 4=बहुत बार, 5=अक्सर, 6=कभी–कभी, 7=कभी नहीं)	19.	☐
20.	Do you feel your family can understand what you are going through? (3= Always; 4= Very often; 5= Quite often; 6= Seldom; 7= Never) क्या आपको लगता हैं कि आपका परिवार यह समझ सकता है कि आप पर क्या बीत रही है? (3=हमेशा, 4=बहुत बार, 5=अक्सर, 6=कभी–कभी, 7=कभी नहीं)	20.	☐

अध्ययन के दौरान गर्भवती न होने के कारण विंगस से बाहर हुई महिलाओं की आधारभूत विशेषताएं, गर्भधारण में सफल न होने के संबंध मे महिलाओं द्वारा किये गये अनुभव और उपाय।

Infertility Study ID: ☐☐☐☐☐☐☐☐☐☐

21.	Have fertility problems strengthened your commitment to your husband?<br>(3= An extreme amount; 4= Very much; 5= A moderate amount; 6= Little; 7= Not at all)<br>क्या आपकी इस (प्रजननक्षमता की) समस्या आपके पति के साथ आपकी प्रतिबद्धता (साथ निभाने का वादा) को और मजबूत किया है ?<br>(3=अत्याधिक मात्रा में, 4=काफी हद तक, 5=ठिक-ठाक मात्रा में, 6=थोड़ी मात्रा मे, 7=बिल्कुल नहीं)	21.	☐
22.	Do you feel sad and depressed because of your fertility problems?<br>(3= An extreme amount; 4= Very much; 5= A moderate amount; 6= Little; 7= Not at all)<br>क्या आप अपनी इस (प्रजननक्षमता की) समस्या की वज़ह से दुखी या उदास महसूस करते है ?<br>(3=अत्याधिक मात्रा में, 4=काफी हद तक, 5=ठिक-ठाक मात्रा में, 6=थोड़ी मात्रा मे, 7=बिल्कुल नहीं)	22.	☐
23.	Do your fertility problems make you inferior to people with children?<br>(3= An extreme amount; 4= Very much; 5= A moderate amount; 6= Little; 7= Not at all)<br>क्या इस (प्रजननक्षमता की) समस्या के कारण आप नीचा महसूस करते है उन लोगों के सामने जिनका बच्चा हैं ?<br>(3=अत्याधिक मात्रा में, 4=काफी हद तक, 5=ठिक-ठाक मात्रा में, 6=थोड़ी मात्रा मे, 7=बिल्कुल नहीं)	23.	☐
24.	Are you bothered by fatigue because of fertility problems?<br>(3= An extreme amount; 4= Very much; 5= A moderate amount; 6= Little; 7= Not at all)	24.	☐

अध्ययन के दौरान गर्भवती न होने के कारण विंगस से बाहर हुई महिलाओं की आधारभूत विशेषताएं, गर्भधारण में सफल न होने के संबंध मे महिलाओं द्वारा किये गये अनुभव और उपाय।

Infertility Study ID: ☐☐☐☐☐☐☐☐☐☐

	इस (प्रजननक्षमता की) समस्या के कारण क्या आपको थकावट परेशान करती है? (3=अत्याधिक मात्रा में, 4=काफी हद तक, 5=ठिक–ठाक मात्रा में, 6=थोड़ी मात्रा मे, 7=बिल्कुल नहीं)		
25.	Have fertility problems had a negative impact on your relationship with your husband? (3= An extreme amount; 4= Very much; 5= A moderate amount; 6= Little; 7= Not at all) क्या आपकी इस (प्रजननक्षमता की) समस्या आपके पति के साथ संबंध में खराब किया हैं (नकारात्मक प्रभाव लाई है)? (3=अत्याधिक मात्रा में, 4=काफी हद तक, 5=ठिक–ठाक मात्रा में, 6=थोड़ी मात्रा मे, 7=बिल्कुल नहीं)	25.	☐
26.	Do you find it difficult to talk to your husband about your feelings related to infertility? [(3= An extreme amount; 4= Very much; 5= A moderate amount; 6= Little; 7= Not at all) इस (प्रजननक्षमता की) समस्या से संबंधित समस्या के बारे मे अपने पति से बात करना मुश्किल लगता है? (3=अत्याधिक मात्रा में, 4=काफी हद तक, 5=ठिक–ठाक मात्रा में, 6=थोड़ी मात्रा मे, 7=बिल्कुल नहीं)	26.	☐
27.	Are you content with your relationship even though you have fertility problems? (3= An extreme amount; 4= Very much; 5= A moderate amount; 6= Little; 7= Not at all) इस (प्रजननक्षमता की) समस्या होने के बावजूद क्या आप पति के साथ रिश्ते से संतुष्ट है?	27.	☐

अध्ययन के दौरान गर्भवती न होने के कारण विंगस से बाहर हुई महिलाओं की आधारभूत विशेषताएं, गर्भधारण में सफल न होने के संबंध मे महिलाओं द्वारा किये गये अनुभव और उपाय।

Infertility Study ID: ☐☐☐☐☐☐☐☐☐☐

	(3=अत्याधिक मात्रा में, 4=काफी हद तक, 5=ठिक-ठाक मात्रा में, 6=थोड़ी मात्रा मे, 7=बिल्कुल नहीं)		
28.	Do you feel social pressure on you to have (or have more) children?<br>(3= An extreme amount; 4= Very much; 5= A moderate amount; 6= Little; 7= Not at all)<br>क्या आप सामाजिक दबाव महसूस करती हैं बच्चा (या एक और बच्चा) करने के लिए?<br>(3=अत्याधिक मात्रा में, 4=काफी हद तक, 5=ठिक-ठाक मात्रा में, 6=थोड़ी मात्रा मे, 7=बिल्कुल नहीं)	28.	☐
29.	Do your fertility problems make you angry?<br>(3= An extreme amount; 4= Very much; 5= A moderate amount; 6= Little; 7= Not at all)<br>क्या इस (प्रजननक्षमता की) समस्या आपको गुस्सा दिलाती है?<br>(3=अत्याधिक मात्रा में, 4=काफी हद तक, 5=ठिक-ठाक मात्रा में, 6=थोड़ी मात्रा मे, 7=बिल्कुल नहीं)	29.	☐
30.	Do you feel pain and physical discomfort because of your fertility problems?<br>(3= An extreme amount; 4= Very much; 5= A moderate amount; 6= Little; 7= Not at all)<br>क्या इस (प्रजननक्षमता की) समस्या होने के कारण क्या आप दर्द या शारीरिक बेचैनी महसूस करते है?<br>(3=अत्याधिक मात्रा में, 4=काफी हद तक, 5=ठिक-ठाक मात्रा में, 6=थोड़ी मात्रा मे, 7=बिल्कुल नहीं)	30.	☐

अध्ययन के दौरान गर्भवती न होने के कारण विंगस से बाहर हुई महिलाओं की आधारभूत विशेषताएं, गर्भधारण में सफल न होने के संबंध मे महिलाओं द्वारा किये गये अनुभव और उपाय।

Infertility Study ID: ☐☐☐☐☐☐☐☐☐☐

Module 3: ACTIONS TAKEN कदम उठाए गये क्रिया

Administer this questionnaire to women who did not conceive in the 18 months of follow up in WINGS

इस प्रश्नावली को उन महिलाओं के लिए भरा जाएगा जो पिछ्ले 18 महिनों में विंगस में फॅलो–अप करते समय गर्भवती नही हुई

[Code: 1=Yes, 2=No, 8=Does not know, 9=Not Applicable]
[कोडः 1=हॉं, 2=नहीं, 8=पता नहीं, 9=मान्य नहीं]

1.	WINGS ID विंगस आई.डी.	1.	☐☐☐☐☐☐
2.	Respondent's study ID number (eg INFERT0001 TO INFERT1530)<br>उतरदेने वाले का स्टडी आई.डी. नम्बर (eg INFERT0001 TO INFERT1530)	2.	☐☐☐☐☐☐☐☐☐☐
3.	Questionnaire administered by प्रश्नावली किसके द्वारा भरा गया	3.	____________________ ☐☐
4.	Consent obtained? सहमति प्राप्ती हुई ?	4.	☐
5.	Date तारीख	5.	☐☐☐☐☐☐☐☐
**MODULE 3: ACTIONS TAKEN**			
6.	Have you sought any help because you did not become pregnant during your participation WINGS?<br>उस परियोजना में शामिल होने के दौरान आप गर्भवती नहीं हुए थेए इस कारण क्या आपने किसी प्रकार की कोई मदद ली हैं गर्भवती होने के लिए क्योंकि आप विंगस में शामिल होने के दौरान गर्भवती नही हो पाए ?	6.	☐
	If YES, what help seeking actions have you taken and how much did you spend over the 18 months you were enrolled in WINGS? (fill all that apply, multiple actions possible)<br>अगर हॉं तो क्या मदद लिया आपने गर्भवती होने के लिए पिछ्ले 18 महीनों में जब आप विंगस में शामिल थी, तब कितना खर्चा हो गया आपका मदद लेने के कारण ?		

अध्ययन के दौरान गर्भवती न होने के कारण विंगस से बाहर हुई महिलाओं की आधारभूत विशेषताएं, गर्भधारण में सफल न होने के संबंध मे महिलाओं द्वारा किये गये अनुभव और उपाय।

Infertility Study ID: ☐☐☐☐☐☐☐☐☐☐

7. Action sought मदद लिया	8. How many of each consulted कितनो के पास गए	9. Number of times कितनी बार लिया	10. Treatment received (11= Seeking advice from a medical doctor (consultation); 12= Undergoing fertility diagnostic testing (diagnosis); 13= Ovulation induction; 14= Insemination; 15= Surgery or; 16= Assisted Reproduction Technology (ART); 17= Drugs; 18= Counselling; 19= None of these; 20=Others) क्या उपचार प्राप्त किया (11= डाक्टर से सलाह ली, 12= बच्चा ना होने के कारण जो जाँचें हैं वो हो रहे हैं, 13= अंडे बनने के लिए कोई इलाज, 14= आदमी के शरीर में जिस चीज़ से बच्चे बनते हैं (वीर्य) वह आपके शरीर में डाला गया हैं, 15= ऑप्रेशन, 16= प्रजनन तकनीक (टेस्ट टयूब) की सहायता ली, 17= दवाईयां, 18= सलाह, 19= इनमें से कोई नही, 20= अन्य)	11. How much have you had to spend on services to assist you conceive over the last 18 months that you were enrolled in WINGS? इस परियोजना में शामिल होने के दौरान पिछले 18 महीने में आपको कितना पैसा खर्चा करना पड़ा गर्भधारण की सेवाओं के लिए
Traditional doctor देसी डाक्टर				
General doctor सामान्य डाक्टर				
Specialist doctor विशेषज्ञ चिकित्सक				
Parent (s) माता या पिता या दोनों				
Family members (non-parent) माता–पिता के अलावा परिवार के कोई और सदस्य				
Religious leader (Prayer or medication) धर्मिक नेता (प्रर्थाना या दवाई के लिए)				

अध्ययन के दौरान गर्भवती न होने के कारण विंगस से बाहर हुई महिलाओं की आधारभूत विशेषताएं, गर्भधारण में सफल न होने के संबंध मे महिलाओं द्वारा किये गये अनुभव और उपाय।

Infertility Study ID:

Accessed a help line कोई हेल्पलाईन से मदद ली				
Non-governmental organisation गैर सरकारी संस्थान				
Government social services सरकारी सामाजिक सेवाओं				
Friend एक दोस्त				
Husband पति				
Joined a peer support group एक सहकर्मी समूह में शामिल हुई				
Did not seek any help काई मदद नही ली				
Government Health system सरकारी स्वास्थ्य संस्था/कार्यकर्ता				
Study staff परियोजना के कर्मचारी				
Other अन्य				

अध्ययन के दौरान गर्भवती न होने के कारण विंगस से बाहर हुई महिलाओं की आधारभूत विशेषताएं, गर्भधारण में सफल न होने के संबंध मे महिलाओं द्वारा किये गये अनुभव और उपाय।

Infertility Study ID: ☐☐☐☐☐☐☐☐☐☐

12.	Where did you get money to pay for the services? आपको कहाँ से पैसे मिले सेवाओं का भुगतान करने के लिए	12.	
12.1	Insurance बीमा	12.1	☐
12.2	Employer नियोक्ता	12.2	☐
12.3	Borrowed from a bank बैंक से उधार लिए	12.3	☐
12.4	Borrowed from a relative or friend. संबंधी या दोस्त से उधार लिए	12.4	☐
12.5	Financial support from parents अपने माता–पिता से पैसे की मदद	12.5	☐
12.6	Others, specify _______________ अन्य, स्पष्ट करें	12.6	☐
13.	How much did you spend on food in the **last month**? पिछले महीने में आपके परिवार ने खाद्य पदार्थ पर कितना खर्च किया था?	13.	☐☐☐☐☐☐
14.	How much did you spend on household utilities in the **last month**? पिछले महीने में आपके परिवार ने घरेलू सामान पर कितना खर्च किया था?	14.	☐☐☐☐☐☐
15.	How much is your total household income **per month**? _______ आपका कुल घरेलू आय एक महीने का कितना हैं?	15.	☐☐☐☐☐☐
16.	If you did NOT seek help, which of the following reasons best describes why not? (spontaneous 3; prompted with probe 4) choose the most important reason) अगर मदद नहीं ली तो क्यों नही ली उसका कारण आप निम्नलिखित से बताएं? (3=अपने आप बताया, 4=पूछने के बाद बताया)	16.	☐

अध्ययन के दौरान गर्भवती न होने के कारण विंगस से बाहर हुई महिलाओं की आधारभूत विशेषताएं, गर्भधारण में सफल न होने के संबंध मे महिलाओं द्वारा किये गये अनुभव और उपाय।

Infertility Study ID: ☐☐☐☐☐☐☐☐☐☐

16.1	I didn't think I had a problem क्या इसलिए आपने ईलाज नहीं करवाया की आपको लगता है कि आपमें कोई समस्या नहीं हैं	16.1	☐
16.2	I was not aware of existence or possibility of fertility treatment क्या इसलिए आपने ईलाज नहीं करवाया की आपको पता नहीं था की इस समस्या का कोई ईलाज हैं	16.2	☐
16.3	I was concerned about what my friends might think, say or do मैं चिन्तित थी की मेरे दोस्त क्या सोच सकते हैं, बोल सकते है या कह सकते हैं	16.3	☐
16.4	I was concerned about what my husband might think, say or do मैं चिन्तित थी की मेरे पति क्या सोच सकते हैं, बोल सकते है या कह सकते हैं	16.4	☐
16.5	I had no one who could help me get professional care मेरा कोई नहीं है जो मुझे मदद करें पेशेवर देखभाल पाने में	16.5	☐
16.6	I wanted to solve the problem on my own मैं खुद अपनी संकट का हल निकालना चाहती हूँ	16.6	☐
16.7	I was concerned that I might be seen as weak for having in infertility problem मैं चिन्तित थी की प्रजननक्षमता न होने के कारण मैं कमजोर के रूप में देखी जा सकती हूँ	16.7	☐
16.8	I had problems with transport or travelling to appointments नियुक्ति के लिए परिवाहन की समस्या होती हैं	16.8	☐
16.9	I thought the problem would get better by itself and I would get pregnant मैनें सेाचा प्रजननक्षमता की समस्या अपने आप ठीक हो जाएगी ओर मै गर्भवती हो जाउंगी	16.9	☐

अध्ययन के दौरान गर्भवती न होने के कारण विंगस से बाहर हुई महिलाओं की आधारभूत विशेषताएं, गर्भधारण में सफल न होने के संबंध मे महिलाओं द्वारा किये गये अनुभव और उपाय।

Infertility Study ID: ☐☐☐☐☐☐☐☐☐☐

16.10	I was concerned about what my family might think, say, do or feel मैं चिन्तित थी की मेरे परिवार वाले क्या सोचेंगें, बोलेंगें, करेंगें या महसूस करेंगें	16.10	☐
16.11	I felt embarrassed or ashamed मझे शार्मिंदर्गी महसूस हुई	16.11	☐
16.12	I preferred to get alternative forms of care क्या आपने इसलिए ईलाज नहीं करवाया की आपको लगा कोई और उपाय या तरीका अपनाओगें	16.12	☐
16.13	I was not being able to afford the financial costs involved जो भी आर्थिक खर्च शामिल था मैं वह जुटा नही पा रही थी	16.13	☐
16.14	I was concerned that I might be seen as not valuable क्या आपने इसलिए ईलाज नहीं करवाया की आपको यह लगता है कि ईलाज करवाने से आपका महत्व कम हो जाएगा।	16.14	☐
16.15	I thought that professional care probably would not help क्या आपने इसलिए ईलाज नहीं करवाया क्योंकि आपको लगा की पेशेवर ईलाज की इस समस्या में कोई फायदा नहीं होगा	16.15	☐
16.16	I was too unwell to ask for help मैं बहुत अस्वस्थ थी इसलिए मदद नहीं मांग पाई	16.16	☐
16.17	I was concerned that people I know might find out about my infertility problem मैं चिन्तित थी की लोग मेरी प्रजननक्षमता की समस्या के बारे मे जान जाएंगें	16.17	☐
16.18	I dislike talking about my feelings, emotions or thoughts मुझे मेर भावनाओं और विचारों के बारे में बात करना पसंद नही हैं	16.18	☐

अध्ययन के दौरान गर्भवती न होने के कारण विंगस से बाहर हुई महिलाओं की आधारभूत विशेषताएं, गर्भधारण में सफल न होने के संबंध मे महिलाओं द्वारा किये गये अनुभव और उपाय।

Infertility Study ID: ☐☐☐☐☐☐☐☐☐☐

16.19	I was concerned that people might not take me seriously if they found out I was having infertility मैं चिन्तित थी की लोग मुझसे गंभीरता के साथ पेश नहीं आएंगें अगर उन्हे मेरी प्रजननक्षमता की समस्या के बारे मे पता चलेगा	16.19	☐
16.20	I was concerned about the treatments available (e.g. medication side effects) मैं उपचारों की उपलबधि के बारे में चिन्तित थी (जैसे कि दवाईयों के दुष्प्रभाव)	16.20	☐
16.21	I had already had previous bad experiences with infertility health care providers प्रजननक्षमता की स्वास्थ्य देखभाल करने वालों के साथ मेरा पहला खराब अनुभव हो चुका था	16.21	☐
16.22	I preferred to get help from family or friends अपने परिवारवालों या दोस्तों से मदद लेना मैनें पसंद किया	16.22	☐
16.23	I preferred traditional help पारम्पारिक सहयता मैने पसंद किया	16.23	☐
16.24	I heard it is very expensive मैने सुना हैं बहुत महंगा हैं	16.24	☐
16.25	I did not have the money मेरे पास पैसे नहीं थे	16.25	☐
16.26	I heard these treatments are for long duration and not effective मैने सुना हैं यह सारे उपचार लम्बी अवधि के लिए है और प्रभावी भी नहीं हैं	16.26	☐
16.27	I know problem is with my husband and he will not seek care मुझे पता है कि समस्या मेरे पति मे हैं ओर वो इसका उपचार नही कराएंगें	16.27	☐
16.28	Other reason, please describe अन्य कारण, कृपया करके वर्णन करें________________	16.28	☐

अध्ययन के दौरान गर्भवती न होने के कारण विंगस से बाहर हुई महिलाओं की आधारभूत विशेषताएं, गर्भधारण में सफल न होने के संबंध मे महिलाओं द्वारा किये गये अनुभव और उपाय।

Infertility Study ID: ☐☐☐☐☐☐☐☐☐☐

17.	Prior to your participation in WINGS had you consulted a doctor to assist you to conceive? उस परियोजना में शामिल होने से पहले क्या आपने कोई चिकित्यक से सहयता ली है जो आपको गर्भवती होने में सहायता करें	17.	☐
18.	Which other actions did you take (fill all that apply, multiple actions possible) आपने और क्या कार्यवाही की (कदम उठाए) है (निम्नलिखित मे से चुने, एक से अधिक चुनने का अधिकार हैं)	18.	
18.1	Had sex more regularly नियमित रूप से यौन क्रिया जारी रखी (शारीरिक संबंध)	18.1	☐
18.2	Used herbal medicines जड़ीबूटी वाली दवा का प्रयोग किया	18.2	☐
18.3	Went to faith healers कोई दाई या झाड़–फूक वाले के पास गये	18.3	☐
18.4	Kept fast and worshipped God उपवास किया और भगवान की पूजा की	18.4	☐
18.5	Douched the vagina अपनी यौनी को अच्छे से साफ किया/खंगाला	18.5	☐
18.6	Others, please describe अन्य कारण, कृपया करके वर्णन करें________________	18.6	☐
19.	Do you know of any place where you could obtain assisted reproduction services to get pregnant? क्या आप कोई ऐसी जगह जानते है जहॉं आपको प्रजनन तकनीक की सेवा (सहायता से) मिलेगी गर्भवती होने के लिए ?	19.	☐
20.	Has your husband sought help from a doctor to increase your chances of getting pregnant? क्या आपके पति ने किसी चिक्त्सिक से मदद मॉंगी है ताकि आपका गर्भवती होने की संभावना बढ़े ?	20.	☐

अध्ययन के दौरान गर्भवती न होने के कारण विंगस से बाहर हुई महिलाओं की आधारभूत विशेषताएं, गर्भधारण में सफल न होने के संबंध मे महिलाओं द्वारा किये गये अनुभव और उपाय।

Infertility Study ID: ☐☐☐☐☐☐☐☐☐☐

21.	Has your husband sought help from family members to increase your chances of getting pregnant? क्या आपके पति ने कोई परिवार वालों से मदद माँगी है ताकि आपका गर्भवती होने की संभावना बढ़े?	21.	☐
22.	Do you think your husband may have a problem that is reducing your chances of getting pregnant? क्या आपको लगता हैं कि आपके पति को कोई समस्या हैं जिसके कारण आपको गर्भवती होने की संभावना कम हो रही हैं?	22.	☐
23.	Is your husband willing to seek advise क्या आपके पति सलाह लेने के लिए तैयार हैं?	23.	☐
24.	If not why<br>अगर नही तो क्यों	24.	
24.1	He has too much ego पति मे बहुत अहंकार हैं	24.1	☐
24.2	He does not believe that even men can have problems वो विश्वास नहीं करते की मर्द को भी कोई समस्या हो सकती हैं	24.2	☐
24.3	He feels shy and ashamed वो संकोच महसूस करते हैं और शार्मिन्दा हैं	24.3	☐
24.4	He is convinced that problem is with me वो यह यकिन करते है कि समस्या मुझमे हैं	24.4	☐
24.5	He is happy not having a baby वो खुश हैं कि बच्चा नही होता	24.5	☐
24.6	He has extramarital affair क्या आपके पति का शादी के बाद किसी और के साथ कोई संबंध हैं	24.6	☐
24.7	He does not have the money उनके पास उपचार के पैस नही हैं	24.7	☐

अध्ययन के दौरान गर्भवती न होने के कारण विंगस से बाहर हुई महिलाओं की आधारभूत विशेषताएं, गर्भधारण में सफल न होने के संबंध मे महिलाओं द्वारा किये गये अनुभव और उपाय।

Infertility Study ID: ☐☐☐☐☐☐☐☐☐☐

24.8	He will have an excuse to remarry यह समस्या एक बहाना है उनके लिए ताकि वह दूसरी शादी कर सके	24.8	☐
24.9	Others अन्य	24.9	☐

अध्ययन के दौरान गर्भवती न होने के कारण विंगस से बाहर हुई महिलाओं की आधारभूत विशेषताएं, गर्भधारण में सफल न होने के संबंध मे महिलाओं द्वारा किये गये अनुभव और उपाय।

Infertility Study ID: ☐☐☐☐☐☐☐☐☐☐

Form D: Module 4

Administer this questionnaire to women who did not conceive in the 18 months of follow up in WINGS

Form D: Module 4

मॉडयूल 4

इस प्रश्नावली को उन महिलाओं के लिए भरा जाएगा जो पिछले 18 महिनों में विंगस में फॅलो–अप करते समय गर्भवती नही हुई

[Code: 1=Yes, 2=No, 8=Does not know, 9=Not Applicable]
[कोडः 1=हाँ, 2=नहीं, 8=पता नहीं, 9=मान्य नहीं]

1.	WINGS ID<br>विंगस आई.डी.	1.	☐☐☐☐☐☐
2.	Respondent's study ID number (eg INFERT0001 TO INFERT1530)<br>उतरदेने वाले का स्टडी आई.डी. नम्बर (eg INFERT0001 TO INFERT1530)	2.	☐☐☐☐☐☐☐☐☐☐
3.	Questionnaire administered by प्रश्नावली किसके द्वारा भरा गया	3.	____________________ ☐☐
4.	Consent obtained? सहमति प्राप्ती हुई ?	4.	☐
5.	Date तारीख	5.	☐☐☐☐☐☐☐☐
**MODULE 4: MEDICAL HISTORY**<br>**मॉडयूल 4ः चिकित्या का इतिहास**<br>**Obstetric and Gynaecological History**<br>**प्रसूति और स्त्री रोग संबंधित इतिहास**			
6.	How many times have you been pregnant?<br>आप कितनी बार गर्भवती हुई हैं ?	6.	☐

अध्ययन के दौरान गर्भवती न होने के कारण विंगस से बाहर हुई महिलाओं की आधारभूत विशेषताएं, गर्भधारण में सफल न होने के संबंध मे महिलाओं द्वारा किये गये अनुभव और उपाय।

Infertility Study ID: ☐☐☐☐☐☐☐☐☐☐

7.	Have you had Miscarriages?<br>क्या कभी गर्भपात हुआ हैं ?	7.	☐
8.	Have you ever had still birth?<br>क्या आपने कभी मृत शिशु को जन्म दिया था ?	8.	☐
9.	Have you had any complications after delivery?<br>क्या आपको प्रसव के बाद कोई परेशानी हुई थी ?	9.	☐
10.	How many abortions have you ever had?<br>अबतक आपके कितने गर्भपात हुए हैं ?	10.	☐
11.	How old were you when your first period started? (years of age)<br>आपकी उम्र क्या थी जब आपको पहली बार मासिक हुआ ? (उम्र के साल)	11.	☐☐
12.	When was the first day of your last period?<br>आपका आखिर मासिक का पहला दिन कब था ?	12.	☐☐☐☐☐☐☐☐
13.	Do you have a period every month?<br>क्या आपको हर महिने मासिक होता हैं ?	13.	☐
14.	Do you have irregular periods, more than once in a month<br>क्या आपका मासिक अनियमित हैं, महिने में एक बार से ज्यादा ?	14.	☐
15.	How many days of gap from the last period to the next one? (days)<br>पिछले और अगले मासिक के बीच कितने दिन का अंतर रहता हैं ? (दिन एक हिसाब से)	15.	☐☐
16.	How many days do your periods last? (days)_________<br>कितने दिन तक मासिक रहता हैं ? (दिन के हिसाब से)	16.	☐☐
17.	Is your bleeding heavy (e.g., are there blood clots)	17.	☐

अध्ययन के दौरान गर्भवती न होने के कारण विंगस से बाहर हुई महिलाओं की आधारभूत विशेषताएं, गर्भधारण में सफल न होने के संबंध मे महिलाओं द्वारा किये गये अनुभव और उपाय।

Infertility Study ID: ☐☐☐☐☐☐☐☐☐☐

	क्या भारी रक्तस्त्राव होता हैं मासिक के दौरान? (क्या खून के टुकड़े निकलते हैं)		
18.	Do you have painful periods?<br>क्या मासिक पीड़ादायक रहता हैं?	18.	☐
19.	Do you have bleeding between your periods?<br>क्या आपका मासिकों के बीच में रक्तस्त्राव होता हैं?	19.	☐
20.	Have you ever used contraception before?<br>क्या आपने पहले कभी गर्भनिरोधक का उपयोग किया हैं?	20.	☐
**Sexual History**<br>यौन इतिहास			
21.	Have you had abnormal or smelly vaginal discharge?<br>क्या कभी आपको अस्वभाविक या बदबूदार यौनी स्त्राव (नीचे से बदबूदार पानी गया हैं) हुआ हैं?	21.	☐
22.	If yes, are you having those symptoms currently?<br>यदि हॉं, तो क्या वर्तमान स्थिति में वह सभी लक्षण हैं?	22.	☐
23.	Have you ever been treated or admitted to hospital for vaginal discharge?<br>क्या आपको कभी यौनी स्त्राव के लिए कारण अस्पताल में ईलाज मिला हैं या अस्पताल मे लिए भर्ती हुए थे?	23.	☐
24.	Have you ever been diagnosed for any of the following conditions? Check records (जॉंच में) क्या कभी आपको बताया गया हैं कि आपको यह बीमारी हैं जैसे कि (रिकॉर्ड चैक करें)	24.	
24.1	Pelvic inflammatory disease (PID) यौन अंगो की बीमारी	24.1	☐

अध्ययन के दौरान गर्भवती न होने के कारण विंगस से बाहर हुई महिलाओं की आधारभूत विशेषताएं, गर्भधारण में सफल न होने के संबंध मे महिलाओं द्वारा किये गये अनुभव और उपाय।

Infertility Study ID: ☐☐☐☐☐☐☐☐☐☐

24.2<br>24.3<br>24.4<br>24.5<br>24.6<br>24.7<br>24.8<br>24.9<br>24.10<br>24.11<br>24.12	Gonorrhoea गनोरिया<br>Chlamydia केलेमायडिन<br>Trichomoniasis<br>Genital herpes जनन अंगों का दाद<br>Syphilis सिफिलिस<br>Polycystic ovary syndrome (PCOS)? अंडाश्य के रोग लक्षण<br>Thyroid disease थॉयरॉयड की बीमारी<br>Hypertension हाई बल्ड प्रेशर<br>Diabetes मधूमेह<br>Any other chronic medical condition including cancer? कोई ओर लम्बे समय तक चलने वाली बीमारी जैसे की कैंसर ?<br>Others, specify_______________ अन्य, स्पष्ट करें	24.2<br>24.3<br>24.4<br>24.5<br>24.6<br>24.7<br>24.8<br>24.9<br>24.10<br>24.11<br>24.12	☐<br>☐<br>☐<br>☐<br>☐<br>☐<br>☐<br>☐<br>☐<br>☐<br>☐
25.	Do you have significant pain during intercourse?<br>क्या आपको शारीरिक मिलन के दौरान बहुत दर्द होता हैं ?	25.	☐
26.	Do you use lubricants during intercourse?<br>क्या आप शारीरिक मिलन के दौरान चिपचिपा पदार्थ का इस्तेमाल करते हैं ?	26.	☐
27.	Do you experience bleeding after intercourse?<br>क्या आपको शारीरिक मिलन के बाद खून निकलता हैं ?	27.	☐
28.	Do you or your husband have any problems during sexual intercourse?	28.	☐

अध्ययन के दौरान गर्भवती न होने के कारण विंगस से बाहर हुई महिलाओं की आधारभूत विशेषताएं, गर्भधारण में सफल न होने के संबंध मे महिलाओं द्वारा किये गये अनुभव और उपाय।

Infertility Study ID: ☐☐☐☐☐☐☐☐☐☐

	क्या आपको या आपके पति को शारीरिक मिलन के दौरान कोई परेशानी होती हैं ?		
29.	Do you feel satisfied with your sex life?<br>क्या आप अपने यौन जीवन से संतुष्ट हैं ?	29.	☐
**Substance use of respondent**<br>उतरदेनेवाले द्वारा मादक द्रवयों का सेवन			
30.	Do you use cigarettes/bidis<br>क्या आप सिगरेट/बीड़ी पीते हैं ?	30.	☐
31.	How many cigarettes do you smoke per day? (#)<br>आप एक दिन में कितनी सिगरेट पीते हैं ?	31.	☐☐
32.	Do you use alcohol?<br>क्या आप शराब लेते हैं ?	32.	☐
33.	Do you use Gutka/pan masal, Khaini?<br>क्या आप गुटका/पान मसाला या खैनी लेते हैं ?	33.	☐
34.	How many days per week do you usually drink alcohol? (#)<br>आमतौर पर हफतें में कितने दिन शराब पीते हैं ?	34.	☐
**Substance use of husband**<br>पति द्वारा मादक द्रवयों का सेवन			
35.	Does your husband use cigarettes/bidis<br>क्या आपके पति सिगरेट/बीड़ी पीते हैं ?	35.	☐
36.	How many cigarettes does your husband smoke per day? (#)<br>आपके पति एक दिन में कितनी सिगरेट पीते हैं ?	36.	☐☐
37.	Does your husband use alcohol?<br>क्या आपके पति शराब लेते हैं ?	37.	☐

अध्ययन के दौरान गर्भवती न होने के कारण विंगस से बाहर हुई महिलाओं की आधारभूत विशेषताएं, गर्भधारण में सफल न होने के संबंध मे महिलाओं द्वारा किये गये अनुभव और उपाय।

Infertility Study ID: ☐☐☐☐☐☐☐☐☐☐

38.	Does your husband use Gutka/pan masal, Khaini?<br>क्या आपके पति गुट्का/पान मसाला या खैनी लेते हैं?	38.	☐
39.	How many days per week does your husband usually drink alcohol? (#)<br>आमतौर पर हफ्तें में कितने दिन आपके पति शराब पीते हैं?	39.	☐
**Mental Health History**<br>मानसिक स्वास्थ्य का इतिहास			
40.	Over the <u>last 2 weeks,</u> how often have you had "little interest or pleasure in doing things" (check only one answer)<br>(3= not at all; 4= several days; 5= more than half the days; 6= nearly everyday)<br>पिछ्ले 2 हफतों में (15 दिन में) आपको ऐसा कितने दिन लगा की कुछ करने में बहुत कम दिलचस्पी या मज़ा आ रहा हो? (केवल एक उत्तर का जाँच करें)<br>(3=बिल्कुल भी नहीं, 4=कई दिन, 5=आधे से ज्यादा दिन, 6=लगभग हर रोज़)	40.	☐
41.	Over the <u>last 2 weeks</u>, how often have you been "feeling down, depressed, or hopeless"?<br>(3= not at all; 4= several days; 5= more than half the days; 6= nearly everyday)<br>पिछ्ले 2 हफतों में (15 दिन में) आपको ऐसा कितने दिन लगा की आप उदास या निराश महसूस कर रही हैं?<br>(3=बिल्कुल भी नहीं, 4=कई दिन, 5=आधे से ज्यादा दिन, 6=लगभग हर रोज़)	41.	☐
42.	Over the <u>last 2 weeks</u>, how often have you felt "Feeling nervous, anxious or on edge?"<br>(3= not at all; 4= several days; 5= more than half the days; 6= nearly everyday)<br>पिछ्ले 2 हफतों में (15 दिन में) आपको ऐसा कितनी	42.	☐

अध्ययन के दौरान गर्भवती न होने के कारण विंगस से बाहर हुई महिलाओं की आधारभूत विशेषताएं, गर्भधारण में सफल न होने के संबंध मे महिलाओं द्वारा किये गये अनुभव और उपाय।

Infertility Study ID: ☐☐☐☐☐☐☐☐☐☐

	बार लगा की आप "बेचैन, चिन्तित हुए या घबराया" महसूस कर रही है ?<br>(3=बिल्कुल भी नहीं, 4=कई दिन, 5=आधे से ज्यादा दिन, 6=लगभग हर रोज़)		
43.	Over the <u>last 2 weeks</u>, how often have you "Not being able to stop or control worrying?<br>(3= not at all; 4= several days; 5= more than half the days; 6= nearly everyday)<br>पिछले 2 हफतों में (15 दिन में) आपको ऐसा कितने बार लगा की आप "चिन्तित होने से रोक नहीं पाए या खुद को नियंत्रित करने में असफल रहें" ?<br>(3=बिल्कुल भी नहीं, 4=कई दिन, 5=आधे से ज्यादा दिन, 6=लगभग हर रोज़)	43.	☐
