# Supplementary material for: Characteristics, experiences and actions taken by women to address delayed conception: A mixed-methods cross-sectional study protocol
Source: PLoS One. 2022 Mar 11;17(3):e0264777. doi: 10.1371/journal.pone.0264777 (PMC8916660; doi:10.1371/journal.pone.0264777)
Supplement: S1 File — (PDF) [file pone.0264777.s001.pdf]

Ethics Review Committee
Centre for Health Research and Development
Society for Applied Studies

45, Kalu Sarai, New Delhi-110016, India

To,

Dr. Sarmila Mazumder
Principal Investigator
Centre for Health Research and Development
Society for Applied Studies
45, Kalu Sarai,
New Delhi-110016, India

Dr. Neeta Dhabhai
Principal Investigator
Centre for Health Research and Development
Society for Applied Studies
45, Kalu Sarai,
New Delhi-110016, India

Ref No: SAS/ERC/RHR-Infertility/2020 Date: 09/JAN/2021

Dear Dr. Mazumder and Dr. Dhabhai,

The Ethics Review Committee reviewed and discussed your application for renewal of approval to conduct the research study titled **"Baseline characteristics, experiences and actions taken by women to address failure to conceive, among women who exit WINGS without getting pregnant"** on 07/JAN/2021.

The following study documents were reviewed:

1. Continuing Review Application Form dated 23/DEC/2020
2. Appendix 1: Proposal Version 3.0 dated 29/APR/2020
3. Appendix 2: Proposal Version 4.0 dated 19/OCT/2020
4. Appendix 3: Letter of approval of amendments dated 30/MAY/2020
5. Appendix 4: Letter of approval of amendments dated 26/OCT/2020
6. Appendix 5: Technical Report 08/DEC/2020

Level of risk	Less than minimal risk	☑	Minimal	☐	More than minimal risk	☐
Type of review	Exempt from review	☐	Expedited	☑	Full committee	☐

The following members of the Ethics Review Committee were present for the telephonic discussion for expedited review meeting held on 07/JAN/2021, 4:00pm at 45, Kalu Sarai, New Delhi – 110 016.

1. Dr. Binoo Nayar, Chairperson
2. Ms. Swati Naik, Acting Member Secretary, Member, Community Representative
3. Ms. Medha Shekhar, Member, Legal Expert
4. Ms. Runa Ghosh, Member, Social Scientist

The members reviewed the technical report and found it satisfactory. They congratulated by team to have achieved the study progress inspite of challenges due to the COVID pandemic.

The total number of study participants approved for administration of quantitative questionnaire: 1530 and around 50 participants for Focus Group Discussion (FGD).

We approve the trial to be conducted in its presented form.

Initial approval date: 10/JAN/2020

Renewal approval date: 09/JAN/2021

Approval expiration date: 08/JAN/2022

Following are the Principal Investigator's Responsibilities with respect to continuous reporting to ERC:

- The Ethics Review Committee expects to be informed about the progress of the study. A status report must be submitted (at least 2 weeks in advance of the approval expiration date) reviewed and approved by the ERC before the current approval expires, or at least annually from the date of this approval, whichever is earlier.
- The PI shall submit / inform (as applicable) any relevant changes that may affect the study in its presented form to ERC for review / notification as applicable, along with relevant documents. Changes or modifications to this research activity must be submitted to and approved by the ERC before they are implemented. Unapproved changes will be considered as protocol deviations and will require reporting to Ethics Committee.
- The PI shall provide a copy of the final study report to the ERC.
- The ERC must be informed of any untoward or serious and unexpected events happening during the course of the study within 24 hours of knowledge of the event by the researcher, followed by follow-up detailed report as and when there are any updates.
- It is the responsibility of the investigator and all research staff to comply with the applicable ethical guidelines governing human subject research.

The ERC is constituted and conforming with the requirements of , ICMR's Ethical Guidelines for Biomedical Research on Human Participants, ICH Harmonised Tripartite Guideline, Guideline for Good Clinical Practice, Declaration of Helsinki. The OHRP Federal Wide Assurance number for ERC is FWA00001757, IRB 00007526.

Yours sincerely,

BINOO NAYAR
Name of Chairperson

Signature of Member Secretary

Signature of Chairperson

Date 09/JAN/2021

Ethics Review Committee
Centre for Health Research and Development
Society for Applied Studies
45, Kalu Sarai, New Delhi-110016, India

To,
Dr. Sarmila Mazumder
Principal Investigator
Centre for Health Research and Development
Society for Applied Studies
45, Kalu Sarai,
New Delhi-110016, India

Dr. Neeta Dhabhai
Principal Investigator
Centre for Health Research and Development
Society for Applied Studies
45, Kalu Sarai,
New Delhi-110016, India

Ref No: SAS/ERC/RHR-Infertility/2020 Date: 30/MAY/2020

Study Title: "**Baseline characteristics, experiences and actions taken by women to address failure to conceive, among women who exit WINGS without getting pregnant"**

Dear Dr. Mazumder and Dr. Dhabhai,

The Ethics Review Committee reviewed and discussed your application to review the above mentioned study documents with amendments on 25/MAY/2020.

The following documents were reviewed:

1. Infertility study proposal version 3.0 dated 29/April/2020 (Track Changes)
2. Infertility study proposal version 3.0 dated 29/April/2020 (Accept Track Changes)
3. Participant information sheet and informed consent form_version 2.0 dated 29/April/2020 (English – Track Changes)
4. Participant information sheet and informed consent form_version 2.0 dated 29/April/2020 (English – Accept Track Changes)
5. Participant information sheet and informed consent form_version 2.0 dated 29/April/2020 (Hindi)
6. Listing of amendments (proposal version 2.0 to version 3.0) dated 29/April/2020
7. Listing of amendments (consent form for women version 1.0 to version 2.0) dated 29/April/2020
8. Translation and back translation certificates

Level of risk	Less than minimal risk	☑	Minimal	☐	More than minimal risk	☐
Type of review	Exempt from review	☐	Expedited	☑	Full committee	☐

The following members of the Ethics Review Committee were present for the telephonic discussion for expedited review meeting held on 25/MAY/2020:

1. Dr. Binoo Nayar, Chairperson
2. Ms. Swati Naik, Acting Member Secretary, Member, Community Representative
3. Ms. Medha Shekhar, Member, Legal Expert
4. Ms. Runa Ghosh, Member, Social Scientist

The study was approved on 10/JAN/2020.

The PI of the study has recently received communication from WHO ERC that has recommended further modifications from the ethical perspective. The WHO ERC stated that the WINGS participants should not feel duress or obligated to participate in the new study, which is likely to happen if they know the new study is linked to WINGS. Hence, it should be explicitly stated to the WINGS participants that this is a separate study. The WHO ERC also advised that the contact details of the WINGS participants should not be shared with the investigators of the new study without their prior consent. To re-emphasize that this is a separate study the WHO ERC recommended that the new study team should contact the women after a gap of 14 to 28 days after they exit from the WINGS. Additionally, a change in the study title was recommended to show that this is not a sub-study.

The required amendments were made in the proposal and in the ICFs.

The Committee agreed that the recommendations made by the WHO ERC are ethically well justified and approved the amended documents.

Initial approval date: 10/JAN/2020

Approval expiration date: 09/JAN/2021

Following are the Principal Investigator's Responsibilities with respect to continuous reporting to ERC:

- The Ethics Review Committee expects to be informed about the progress of the study. A status report must be submitted (at least 2 weeks in advance of the approval expiration date) reviewed and approved by the ERC before the current approval expires, or at least annually from the date of this approval, whichever is earlier.

- The PI shall submit / inform (as applicable) any relevant changes that may affect the study in its presented form to ERC for review / notification as applicable, along with relevant documents. Changes or modifications to this research activity must be submitted to and approved by the ERC before they are implemented. Unapproved changes will be considered as protocol deviations and will require reporting to Ethics Committee.

- The PI shall provide a copy of the final study report to the ERC.

- The ERC must be informed within twenty four (24) hours of occurrence of any serious and unexpected adverse event and a report on relatedness of these within 14 calendar days (For regulated study)./ The ERC must be informed of any untoward or serious and unexpected events happening during the course of the study (For non-regulated study)

It is the responsibility of the investigator and all research staff to comply with the applicable ethical guidelines governing human subject research.

The ERC is constituted and conforming with the requirements of ICMR's Ethical Guidelines for Biomedical Research on Human Participants, ICH Harmonised Tripartite Guideline, Guideline for Good Clinical Practice and Declaration of Helsinki. The OHRP Federal Wide Assurance number for ERC is FWA00001757, IRB 00007526.

Yours sincerely,

BINOO NAYAR
Name of Chairperson

Signature of Member Secretary

Signature of Chairperson

Date: 30/MAY/2020

Ethics Review Committee
Centre for Health Research and Development
Society for Applied Studies
45, Kalu Sarai, New Delhi-110016, India

To,
Dr. Sarmila Mazumder
Principal Investigator
Centre for Health Research and Development
Society for Applied Studies
45, Kalu Sarai,
New Delhi-110016, India

Dr. Neeta Dhabhai
Principal Investigator
Centre for Health Research and Development
Society for Applied Studies
45, Kalu Sarai,
New Delhi-110016, India

Ref No: SAS/ERC/RHR-Infertility/2020 Date: 26/OCT/2020

Study Title: "**Baseline characteristics, experiences and actions taken by women to address failure to conceive, among women who exit WINGS without getting pregnant"**

Dear Dr. Mazumder and Dr. Dhabhai,

The Ethics Review Committee reviewed and discussed your application to review the above mentioned study documents with amendments on 23/OCT/2020.

The following documents were reviewed:

1. Infertility study proposal version 4.0 dated 19/Oct/2020 (Track Changes)
2. Infertility study proposal version 4.0 dated 19/Oct2020 (Accept Track Changes)
3. Listing of amendments (proposal version 3.0 to version 4.0) dated 19/Oct/2020

Level of risk	Less than minimal risk	☑	Minimal	☐	More than minimal risk	☐
Type of review	Exempt from review	☐	Expedited	☑	Full committee	☐

The following members of the Ethics Review Committee were present for the telephonic discussion for expedited review meeting held on 23/OCT/2020:

1. Dr. Binoo Nayar, Chairperson
2. Ms. Swati Naik, Acting Member Secretary, Member, Community Representative
3. Ms. Medha Shekhar, Member, Legal Expert
4. Ms. Runa Ghosh, Member, Social Scientist

The study was approved on 10/JAN/2020.

Considering the delay in study initiation due to COVID 19, the committee agreed to the proposed strategy to contact women who exited the main WINGS beyond 14 days and not restrict to the earlier strategy of contacting women 14 to 28 days of exit from WINGS. This amendment has no ethical implications, rather the longer gap between the exit from WINGS and enrolment into the new study will be a better justification and strengthen the rationale from the ethical perspective. It is more likely that women will consider this as a separate study and will not feel coerced or obliged to participate in the same.

The Committee agreed with the amendment and approved the amended documents.

Initial approval date: 10/JAN/2020

Approval expiration date: 09/JAN/2021

Following are the Principal Investigator's Responsibilities with respect to continuous reporting to ERC:

- The Ethics Review Committee expects to be informed about the progress of the study. A status report must be submitted (at least 2 weeks in advance of the approval expiration date) reviewed and approved by the ERC before the current approval expires, or at least annually from the date of this approval, whichever is earlier.

- The PI shall submit / inform (as applicable) any relevant changes that may affect the study in its presented form to ERC for review / notification as applicable, along with relevant documents. Changes or modifications to this research activity must be submitted to and approved by the ERC before they are implemented. Unapproved changes will be considered as protocol deviations and will require reporting to Ethics Committee.

- The PI shall provide a copy of the final study report to the ERC.

- The ERC must be informed within twenty four (24) hours of occurrence of any serious and unexpected adverse event and a report on relatedness of these within 14 calendar days (For regulated study)./ The ERC must be informed of any untoward or serious and unexpected events happening during the course of the study (For non-regulated study)

It is the responsibility of the investigator and all research staff to comply with the applicable ethical guidelines governing human subject research.

The ERC is constituted and conforming with the requirements of ICMR's Ethical Guidelines for Biomedical Research on Human Participants, ICH Harmonised Tripartite Guideline, Guideline for Good Clinical Practice and Declaration of Helsinki. The OHRP Federal Wide Assurance number for ERC is FWA00001757, IRB 00007526.

Yours sincerely,

BINOO NAYAR
Name of Chairperson

Signature of Member Secretary

Signature of Chairperson

Date: 26/OCT/2020

Ethics Review Committee
Centre for Health Research and Development
Society for Applied Studies
45, Kalu Sarai, New Delhi-110016, India

To,
Dr. Sarmila Mazumder
Principal Investigator
Centre for Health Research and Development
Society for Applied Studies
45, Kalu Sarai,
New Delhi-110016, India

Dr. Neeta Dhabhai
Principal Investigator
Centre for Health Research and Development
Society for Applied Studies
45, Kalu Sarai,
New Delhi-110016, India

Ref No: SAS/ERC/RHR-Infertility/2020 Date: 10/JAN/2020

Dear Dr. Mazumder and Dr. Dhabhai,

The Ethics Review Committee had reviewed your application to conduct the research study titled "**An ancillary study to WINGS: Baseline characteristics, experiences and actions taken by women to address failure to conceive, among women who exit WINGS without getting pregnant"** in a full committee review meeting conducted on 02/JAN/2020.

The following documents were reviewed:

1. Study proposal version 2.0 dated 21st December 2019
2. Listing of amendments of the research study proposal version 1.0 dated 28th November 2019 to version 2.0 dated 21st December 2019
3. Full review form dated 28/Nov/2019
4. Undertaking by the Investigator dated 28/Nov/209
5. Participant information sheet and informed consent forms version 1.0 dated 28/Nov/2019 (English)
6. Participant information sheet and informed consent forms version 1.0 dated 28/Nov/2019 (Hindi)
7. Translation and back translation certificates
8. CVs of the investigators

The committee noted that proposal version 1.0 dated 28/NOV/2019 was submitted earlier to the committee, following which WHO ERC suggested few amendments. The PI made these changes and re-submitted the revised proposal version 2.0 dated 21/DEC/2019, on 23/DEC/2019. These recommendations and amendments were shared by the PI during the full committee review meeting. The committee agreed with and approved the amendments.

Level of risk	Less than minimal risk	☑	Minimal	☐	More than minimal risk	☐
Type of review	Exempt from review	☐	Expedited	☐	Full committee	☑

The following members of the Ethics Review Committee were present at the meeting held on 02/JAN/2020, 3:45pm at 45, Kalu Sarai, New Delhi – 110 016.

1. Dr. Binoo Nayar, Chairperson, Member, Clinician
2. Ms. Swati Naik, Acting Member Secretary, Member, Community Representative
3. Dr. Jeeva Sankar, Member, Basic Medical Scientist
4. Colonel Gian Sagar, Member, Legal Expert
5. Dr. Sanghmitra Acharya, Member, Social Scientist
6. Dr. Satinder Aneja, Member, Clinician (Observer)

We approve the trial to be conducted in its presented form.

Initial approval date: 10/JAN/2020
Approval expiration date: 09/JAN/2021

The total number of study participants approved for administration of quantitative questionnaire: 1530 and around 50 participants for Focus Group Discussion (FGD).

Following are the Principal Investigator's Responsibilities with respect to continuous reporting to ERC:

- The Ethics Review Committee expects to be informed about the progress of the study. A status report must be submitted (at least 2 weeks in advance of the approval expiration date) reviewed and approved by the ERC before the current approval expires, or at least annually from the date of this approval, whichever is earlier.
- The PI shall submit / inform (as applicable) any relevant changes that may affect the study in its presented form to ERC for review / notification as applicable, along with relevant documents. Changes or modifications to this research activity must be submitted to and approved by the ERC before they are implemented. Unapproved changes will be considered as protocol deviations and will require reporting to Ethics Committee.

- The PI shall provide a copy of the final study report to the ERC.
- The ERC must be informed of any untoward or serious and unexpected events happening during the course of the study.

It is the responsibility of the investigator and all research staff to comply with the applicable ethical and/or regulatory guidelines governing human subject research.

The ERC is constituted and conforming with the requirements of ICMR's Ethical Guidelines for Biomedical Research on Human Participants, ICH Harmonised Tripartite Guideline, Guideline for Good Clinical Practice, Declaration of Helsinki. The OHRP Federal Wide Assurance number for ERC is FWA00001757, IRB 00007526.

Yours sincerely,

BINOO NAIR

Name of Chairperson

10.01.2020

Signature of Member Secretary

Signature of Chairperson

10-01-2020.

Date
