# Supplementary material for: Characteristics, experiences and actions taken by women to address delayed conception: A mixed-methods cross-sectional study protocol
Source: PLoS One. 2022 Mar 11;17(3):e0264777. doi: 10.1371/journal.pone.0264777 (PMC8916660; doi:10.1371/journal.pone.0264777)
Supplement: S2 File — (PDF) [file pone.0264777.s002.pdf]

WHO ERC
Review Summary

Protocol ID: A65998
Country: India
Protocol Title: An ancillary study to WINGS: Baseline characteristics, experiences and actions taken by women to address failure to conceive, among women who exit WINGS without getting pregnant.
Version: 1 **Dated:** 26/09/2019
WHO Responsible Staff Member: Daniel Gitau MBURU
Responsible Unit: WHO/HQ/FWC/RHR
Meeting Date: 13/02/2020

Dear Dr. Daniel Gitau MBURU,
Please find the review summary of the Protocol "An ancillary study to WINGS: Baseline characteristics, experiences and actions taken by women to address failure to conceive, among women who exit WINGS without getting pregnant", which was submitted to the Secretariat on 25/11/2019. This proposal underwent regular review.

The outcome of the review is provided below. When responding, please submit the following:

1. A cover memorandum that addresses your responses, POINT BY POINT, to each of the queries in sections A and B.
   Section C contains Suggestions to improve the proposal but there is no obligation to follow them.

2. An Amended protocol including the responses in bold, highlighted or in track changes. Please ensure that tracking formatting changes is switched off or that all formatting changes have been accepted and that no comments which the team may exchange during the editing are included in the track changes version. The protocol should include all relevant documentation (ICF, study instruments, peer review, etc.) even if already submitted.

Please note that comments in the introductory paragraph are meant for the WHO Responsible Staff Member, though you may decide to share them with the PI.

PLEASE RESPOND TO THIS REVIEW SUMMARY WITHIN A three MONTH PERIOD, OR PROVIDE THE ERC SECRETARIAT A VALID JUSTIFICATION FOR THE DELAY.

The ERC considered that this protocol would benefit from further specification on key aspects such as identification of potential participants, the linkages that will be made with health services to facilitate referrals, the potential involvement of men in the study, etc. A major concern was related to the fact that referring to "infertility" in the consent document and study instruments may distress and possible stigmatize participants. Please see specific comments below.

It is noted that a series of studies using the study population from the WINGS study are being planned by the unit. The ERC understands the great opportunity of accessing a representative sample of women -through the work done by the original study, to answer different research questions. However, the committee is concerned about the burden of participation for women. In order to ensure a fair distribution of the benefits and burdens of research, the unit is requested to ensure that *each woman takes part in only one additional study*, and to explain the process that will be followed to ensure that each woman is only approached once about all ancillary studies.

A. Amendments (Response and change required)

This section includes queries and comments on your protocol, study instruments or the informed consent form for which the ERC requires your response and where relevant, appropriate amendments to the protocol, study instruments or the informed consent.

1. Protocol
   1.1. Please provide an amended proposal specifying the version number and/or date on each page.

1.2. The ERC considered this study is unrelated to the WINGS study. The only commonality is that the same study population will be used. Please remove all reference to "sub-study" from the protocol and the consent documents.

1.3. Under "Hypothesis" it is specified "…this study may generate hypothesis …. on socio-economic impacts of infertility". In addition, one of the study outcomes is "costs related to interventions to enhance fertility". Please note that no data related to this topic is being collected, therefore, the claims about the hypothesis and related study components should be removed unless they can be strongly justified.

In relation to potential risks and benefits:

1.4. It is noted that the definition of "infertility" does not imply a diagnosis or even an abnormality, however, participants may not understand this and consider that they have a "problem" which was unknown until they took part in the study. The fact that someone with knowledge states that they are "infertile" or have "fertility problems" may trigger all sort of reactions (i.e. guilt, fear, anxiety, shame, etc.). The wording being used in the protocol, the study instruments, and the consent documents needs to be sensitive to this issue (e.g. reference could be made to *difficulties in conceiving* rather than "infertility").

In relation to fairness:

1.5. Please specify how the study will ensure that an equal proportion of participants from both arms of the WINGS study are selected for the study under review.

1.6. It is specified that field workers from the WINGS study will inform women and seek their consent to take part in the new study on the last day of participation to WINGS. Given that autonomy of women may be affected by their relationship with the WINGS study team, and that it is the responsibility of the new study team to be sure that informed consent has been obtained, it is not appropriate to have WINGS team members consenting women. Members of the WINGS study team should request permission from women to be contacted by the new study team. Alternatively, members of the WINGS study team could hand out written information to women asking to contact the new study team in case they are interested in the study.

1.7. Please note that at the time of the first visit, women should be explained the study and given time to reflect on whether they wish to participate. Therefore, the option of escorting women to the study clinic on the same day that they receive information about the study does not seem appropriate. Please modify.

1.8. Please specify how participants to the focus groups discussions (FGDs) will be selected.

1.9. Since difficulties in conceiving may affect both partners, please explain whether the study intends -in case women are consulted first and agree, to include men in the interviews. Their involvement could help in reducing the psychological burden of women in relation to conception.

In relation to researcher's obligations:

1.10. The study addresses a highly sensitive and potentially distressing topic and requests participants to dedicate time and effort. However, offers no help or treatment in case of emotional distress, physical/psychological abuse, or a possible STI identified. The study should clearly specify the services that will be provided to women, the referral systems that will be implemented to ensure that referrals are effective (i.e. that women can access services), and what will be covered by the study (i.e. transportation to the clinics, medical examinations, etc.).

In relation to privacy and confidentiality:

1.11. Under "Module 1" it is stated that "These data on characteristics of the sample will be complemented by data being collected in WINGS, as it will be possible to access these data for all women who participate in this sub-study, using unique WINGS participant identifiers". Please note that the study team cannot access data collected from women in the context of the WINGS study. In order to access such data, permission should be requested from women and the consent document modified for such purposes.

2. Study Instruments

2.1. It is unclear why the questionnaire does not include questions on whether the husband has sought medical consultation and/or treatment for fertility. Even if it is not culturally expected that men will seek this type of consultation, it will reassure women on the fact that they are not the only ones who may have difficulties conceiving.

3. Informed Consent Forms

3.1. The consent document states "Your part in this research will take about half an hour to respond to our questions", however, under "Module 4" it is stated: "It is estimated that it would take 1.5 to 2 hours to respond to all the four modules". It is noted that the pilot will provide further information on the time required from participants, however, the consent document needs to be aligned with the protocol. Please address.

Under "Purpose of the study":

3.2. Please note that the wording of the consent document should be extremely careful as not to put "labels" on women. Please refer to comment 1.4 above.

3.3. Some reasons for infertility are given, however, none of them are related to male infertility which raises concerns given that it may increment feelings of guilt among women. Please address.

B. Clarifications (Response required but change may not be required)

This section includes queries on your protocol, study instruments or the informed consent form for which the ERC requires to make changes to your protocol.

NIL

C. Suggestions

This section consists of suggestions for alternative scientific or technical approaches or methods for conducting the research but which do not raise critical, ethical issues. These are meant to be helpful to investigators and are presented as suggestions for you to consider incorporating into a revised protocol. No response from you is required for any comment in this section. If, however, you do make changes to the protocol as a result of these suggestions, please submit the revised protocol to the ERC.

NIL

20, AVENUE APPIA – CH-1211 GENEVA 27 – SWITZERLAND – HTTP://WWW.WHO.INT/ETHICS/REVIEW-COMMITTEE/EN/ – HTTPS://EXTRANET.WHO.INT/ERCWEB/LOGIN.PHP

Protocol ID: A65998 **Meeting Date**: 13/02/2020

Based on the above comments, the Committee has the following recommendation(s) for this proposal:

[ ] The proposal is ***Approved as submitted.*** No modifications are required.

[X] The proposal is ***Conditionally Approved; requires amendments and/or clarifications.*** Final approval is contingent upon an adequate response by the Principal Investigator, to the satisfaction of the reviewers or the Chair on behalf of the ERC.

[ ] The proposal is ***Not approved; requires additional information and/or rewriting.*** A revised version of the proposal should be re-submitted by the WHO responsible staff member as a new submission to the ERC for re-review by Committee.

[ ] The proposal is ***Rejected***. The proposal is ethically unacceptable, for the reasons stated above. The Principal Investigator may submit a new proposal that takes into consideration the ethical issues raised by the Committee. If you do not agree with the Committee's assessment, please feel free to submit an appeal to the Chair of the ERC, through the Secretariat.

NOTE: Final Approval of the Proposal is contingent upon submission of the following:

[ ] Local ethics approval(s) [ ] Other relevant documents

The ERC would like to receive a copy of the recommendations of the local ethics committee when available.

IMPORTANT

1. *Any changes to the proposal* *<u>or</u>* *to the attachments (informed consent/study instruments etc.) should be approved by ERC before being implemented.*
2. *The approval for this proposal is valid for a period of* ***one year only.***
3. *Please resubmit this proposal for a Continuing Review at least 2 months before the next re-approval period.*

Chairperson .... **Date** 21.2.2020

Name: Les Olson

**FINAL APPROVAL**	**FOR THE SECRETARIAT**
Amendments and Clarifications to the proposal have been reviewed. The protocol (Version: Date:) and informed consent Forms (Dated:) submitted on .... are approved by the ERC<br><br>**Chairperson** .... **PO/LO/AL**<br>**Name** ....<br>**Date** 07.05.2020..	Amendments and Clarifications to be reviewed:<br>[ ] Electronically by ERC<br>[ ] by Primary reviewers<br>[ ] by Secretariat<br><br>Amendments approved / Clarifications accepted on<br>Local ERC approval(s) obtained on<br><br>Relevant Documents submitted on<br><br>Comments:<br><br>Signature Date

World Health Organization

Research Ethics Review Committee (WHO ERC)

20, Avenue Appia – CH-1211 Geneva 27 – Switzerland — HTTP://INTRANET.WHO.INT/HOMES/RPC/ERC — HTTP://WWW.WHO.INT/RPC/RESEARCH_ETHICS

WHO ERC
Continuing Review Form for Principal Investigator and WHO Responsible Staff Member

*This form along with the required documentation (please see below) should be submitted by the Principal Investigator at least 2 months prior to the expiry of the ERC approval, so that the ethics approval may be maintained in a streamlined manner, and without any interruption. The date of validity of the approval from the ERC has already been notified to the WHO Responsible Officer through an ERC review summary or memorandum. This form **must** be validated by the WHO Responsible Officer.*

Protocol ID: A-ID: A65998 PI Name: Sarmila Mazumder Name of the WHO Responsible Officer: Gitau Mburu

Protocol Title: Baseline characteristics, experiences and actions taken by women to address failure to conceive, among women who exit WINGS without getting pregnant.

Review Period: From 07/05/2020 to 07/05/2021

Please confirm that the following documents are attached with this form:
1. Progress report for the period under review Yes☒
2. DSMB Report (if applicable) Yes☐ N/A☒
3. Requests for Amendments (if required). Yes☐ N/A☒

Please provide the information requested in the table below:

1. Total expected number of participants to be recruited during the entire study period	1530		
2. Total number of participants actually recruited during the entire study period.	1000		
3. Number of participants recruited since last review	1000		
4. Number of participants who withdrew, were not evaluable or were excluded after initial enrolment during the entire study period	51		
5. Who provided oversight to this project?	Human Reproduction Programme (HRP), World Health Organization, Geneva, Switzerland		
6. How many monitoring visits have taken place since the last approval?	0 visits due to COVID, but monthly calls held		
	Yes	No	NA
7. Were all the issues raised during the monitoring visits addressed to the satisfaction of the sponsors?	☒	☐	☐
8. If a DSMB meeting took place in the period under review, were its recommendations complied with?	☐	☐	☒
**Give details here**:			
9. Have there been any changes in the protocol since the last approval period?	☒	☐	☐
**If Yes, give details here: Based on the WHO ERC recommendations, a 14 to 28 days window period was added to the proposal for enrolling women after their exit from WINGS. The WHO ethics committee had recommended that the investigators should ensure that a gap between exit from the main study (WINGS) and enrolment into the new study, is maintained, so that the women understand that the new study is not a part of WINGS, or else they may feel obliged to participate in the new study. The committee suggested that the participants should be enrolled 14 to 28 days after their exit from WINGS.<br>Due to the COVID pandemic and delays in approval, initiation of the study was delayed by almost 2 years.<br>Over these 2 years, enrollment into the WINGS had been completed and very few women were available to achieve the desired sample sizes in the new study. Therefore, it was agreed based on a consensus by the investigators at SAS and WHO, that the only way to complete the study would be to go back in time and contact women who had exited WINGS earlier. This would imply that the gap of 14-28 days will be exceeded.<br>This has no ethical implication, rather with a longer gap between the exit from WINGS and enrolment into the new study, the justification and rationale become stronger. It is more likely that women will consider the new study as separate from WINGS due to the longer gap.**			
10. Has this change been communicated to ERC and been approved?	☒	☐	☐
**If Yes, give details here**: The local ERC approved the amendment as there was no ethical concern. The committee was of the opinion that that the increase in the duration strengthens the initial rationale to sepate the participants from the praent study even further. We have received the local ERC approval, and have attached it here. The consent document did not mention the 14-28 days duration and therefore did not need to be revised.			
11. Has the consent documents been revised?	☐	☒	☐
**If Yes, give details here:**			
12. Have the revised consent documents been approved by ERC?	☐	☐	☒

	Yes	No	NA
13. **Unanticipated Adverse Events**: During this reporting period, did the study have<br>Any study related ***serious*** unanticipated adverse event?<br>Study related anticipated adverse events at a ***higher frequency***<br>Study related anticipated adverse event at a ***severity higher*** than expected.<br>*(This could include physical, psychological or emotional harm to the participants or it could be problems encountered during consent taking or giving. Please do not submit SAE forms unless explicitly asked to do so)*	☐<br>☐<br>☐	☐<br>☐<br>☐	☒<br>☒<br>☒
14. Have these unanticipated adverse events been reported to the ERC?	☐	☐	☒
15. Have any Serious Adverse Events RELATED to the study product or a study procedure occurred since the last ERC approval?	☐	☐	☒
16. If yes,<br>1. please indicate which of the following review bodies have these been reported to<br>☐ local Ethics Committee ☐ Data Safety Committee ☐ Independent Monitoring Committee<br>2. please attach a listing of these events (including type, severity, outcome) and any feedback that you may have received from reviewers			
17. Pregnancy Related concerns:<br>If contraception or pregnancy was a reason for withdrawal from the protocol - Have any pregnancies occurred during that period?<br>If yes, were these followed up as per protocol?<br>If applicable, please attach a listing of these events (including outcome of follow up).	☐<br>☐	☐<br>☐	☒<br>☒
18. Protocol Violations:<br>Were there any protocol violations (i. e. were any procedures performed or activities undertaken which were not planned for in the approved study protocol) in the period under review?<br>Were these violations reported to the sponsor?<br>Were these violations reported to the independent monitor?	☐<br>☐<br>☐	☐<br>☐<br>☐	☒<br>☒<br>☒
19. Care provision and compensation to participants:<br>Are research participants receiving the care and compensation that was approved by the ERC?	☒	☐	☐
20. Did any new findings, knowledge, or adverse effects come to light that should have been communicated to research participants?	☐	☒	☐
21. **If yes, give details here:**			
22. Were these changes communicated to the research participants?	☐	☐	☒
23. If Yes, then explain how these have been communicated:			

PROTOCOL ID: A-ID: A65998

Thank you for completing this form. Please read next page for important information.

Signature of the Principal Investigator Sarmila Mazumder………………………

To be completed by the WHO Responsible Officer: I confirm that I have reviewed the Progress Report, and it has also been reviewed/not been reviewed by a peer review panel. All required documentation is attached.

Signature …………………………..

Name………………………………

Please turn over page

20, Avenue Appia – CH-1211 Geneva 27 – Switzerland — HTTP://INTRANET.WHO.INT/HOMES/RPC/ERC — HTTP://WWW.WHO.INT/RPC/RESEARCH_ETHICS

PROTOCOL ID:

Project Closure:

Please note that the ERC considers a project "closed" for its purposes (even though data analysis and report writing is on-going) when the investigators will no longer need to have any contact with any research participants – either because the project is terminated early, or the recruitment, interventions and follow-up of participants has been completed, AND all samples and data have been de-identified. If identifiable samples or data are being stored for a longer term after the "closure" of a project, this must have been approved by the ERC.

In case you consider that this project fulfils this condition, please fill in the following form. This form should be submitted by the Principal Investigator and counter-signed by the WHO Responsible staff member.

Date of Closure of project:

Reason for closure:
- ☐ Project never initiated (no human subjects were recruited)
- ☐ Project cancelled. Please specify reason:
- ☐ Data collection completed, **as proposed; data analysis ongoing**
- ☐ Data collection completed, **as proposed; data analysis completed.**

Do you think you will achieve your planned objectives? Yes ☐ No ☐
Do you confirm a compliance with ethical issues raised through the ethics review? Yes ☐ No ☐
Please outline your plans for **archival or destruction** of materials, data and /or samples:
Please outline your plan for **dissemination of results** (if different from what was approved):
Please indicate anticipated timeline for dissemination

Do you verify that informed consent was obtained for all subjects and that all signed consent forms are on file?
Yes ☐ No ☐

Thank you for your compliance with the ERC request and recommendations. Please submit a copy of the final report of the project, within a two year time frame.
Please indicate anticipated time for completion
If we do not hear from you, we will send you a reminder 2 years after the date of closure of the project.

Name of the Principal Investigator **Signature ………………………**

Name of the WHO Responsible Staff member **Signature ……………………….**
