# Supplementary material for: Characteristics, experiences and actions taken by women to address delayed conception: A mixed-methods cross-sectional study protocol
Source: PLoS One. 2022 Mar 11;17(3):e0264777. doi: 10.1371/journal.pone.0264777 (PMC8916660; doi:10.1371/journal.pone.0264777)
Supplement: Attachment — Submitted filename: Review.docx [file pone.0264777.s006.docx]

**Minor grammatical errors need correction**

1. Abstract background line number 22 and 23- The sentence is not imparting a clear meaning as to what the author wants to convey.
2. Line number 118 - Modify sentence “women are not be under duress”
3. Line number 134 - All study documents are to be stored (to added)
4. Detailed questionnaire is not attached
